# Time to really share real-world data?

**DOI:** 10.12688/f1000research.15517.1

**Published:** 2018-07-11

**Authors:** Sophie Graham, Laura McDonald, Radek Wasiak, Michael Lees, Sreeram Ramagopalan

**Affiliations:** 1 Real-World Evidence, Evidera, London, W6 8DL, UK; 2 Centre for Observational Research and Data Sciences, Bristol-Myers Squibb, Uxbridge, UB8 1DH, UK; 3 Worldwide Health Economics and Outcomes Research, Bristol-Myers Squibb, Paris, F-92500, France

**Keywords:** real-world data, sharing, clinical data

## Abstract

Data other than that from clinical trials are important for healthcare decision making. However, unlike the vocal calls seen for more open access to trial data, there are limited efforts being made to ensure that agencies that collect real-world data (RWD) share this, despite its importance. There are many RWD sources across the world that could be readily exploited for research once shared. There are policy and privacy questions that need to be tackled, but opening up and sharing RWD offers remarkable potential for improvements in care for individuals and more effective use of limited healthcare resources. Open science should become the standard for RWD as well as clinical trials, especially those that have a high likelihood to influence practice.

## Introduction: Real-world data and its importance

Real-world data (RWD) are data collected in the course of routine health care delivery or otherwise generated without constraints, as in the ‘real-world’ ^1, 2^. These data are used to understand disease epidemiology, patterns of care and patient need as well as provide valuable insight into treatment effectiveness and safety in day to day clinical practice.

Due to their potential to provide insight into questions not effectively addressed from clinical trials, RWD are increasingly influencing health care decision making, including regulatory assessment, clinical practice and policy. For example, the 21 ^st^ Century Cures Act in the United States has required the Food and Drugs Administration (FDA) to draw up guidelines for the role of RWD in drug approvals ( https://healthpolicy.duke.edu/sites/default/files/atoms/files/rwe_white_paper_2017.09.06.pdf), and in the United Kingdom (UK) the Academy of Medical Sciences and the Association of the British Pharmaceutical Industry have recently prioritised supporting the inclusion of RWD in regulatory and health technology assessment (HTA) processes ( https://acmedsci.ac.uk/more/news/next-steps-for-using-real-world-evidence). These data are also influencing policy; for example, in the UK the controversial decision to increase hospital staffing levels on the weekend, was driven by analysis from inpatient National Health Service (NHS) hospital data from 2009–10 showing a significantly higher risk of mortality in the 30-day follow up period from patients admitted on the weekend, compared to those admitted midweek ^3^.

## Why share data?

The benefits of sharing data are evidenced with the increasing transparency of clinical trial data. Access to clinical trial data has allowed the validation of initially reported trial results ^4^, but perhaps more importantly openly available data has also been used to answer new questions about disease. For example, the Dialogue for Reverse Engineering Assessment and Methods (DREAM) challenge aimed at identifying a prognostic model for overall survival in patients with metastatic prostate cancer using the raw data from the comparator arms of four clinical trials led to the identification of aspartate aminotransferase as an important prognostic factor for overall survival ^5^. Similarly, the SPRINT competition challenged entrees to re-analyse data from a randomized trial of blood-pressure control and this led to the development of a new decision-making tool for clinicians to determine whether patients should receive intensive hypertensive treatment or not based on patient characteristics ^6^

In genetics, the sharing of data have been a major enabling factor in the identification of disease associated genes. Genome-wide association studies often require data from tens of thousands of patients to have the statistical power to detect variants implicated in disease ^7^. Initiatives such as the UK Biobank ^8^ have enabled richly genotyped and phenotyped data on many thousands of patients to be available to the international scientific community. In doing so, these data have been used to better understand the genetic architecture underlying a number of complex diseases including Alzheimer’s, major depression and atrial fibrillation, among many others ^8– 10^. Given the often prohibitively high time and cost burden associated with establishing a cohort needed to unpick the genetic underpinnings of disease, the model employed by the UK Biobank exemplifies the benefits of data sharing not only by enabling reproducibility, but critically in the acceleration and advancement of scientific research.

A number of European clinical databases provide access to RWD. For example, in the UK, the Clinical Practice Research Datalink (CPRD), an electronic medical record database that covers general practitioner encounters, has been available to researchers and has led to the generation of hundreds of peer-reviewed publications, with notable contributions including validation of the safety of the measles, mumps and rubella vaccine ^1^. Further, CPRD data are being increasingly used in English national clinical guidelines and guidances ^11^. The Nordic countries also have a long tradition of collecting patient medical information in the form of national population-based and prescription registries. The high quality of the data recorded and the good coverage of the sources has contributed to a vast number of pharmacoepidemiological insights ^12, 13^, for example in realising risks associated with serotonin reuptake inhibitors in pregnancy ^14^.

Combining accessible RWD sets is of particular importance in Post-Authorisation Safety Studies (PASS), which are studies carried out after a medicine has been approved for use to obtain further information on its safety in the real-world. In such studies, large patient numbers are required to increase power to detect true rare adverse outcomes associated with a particular treatment. To meet the FDA’s Post Marketing Requirements for a post-marketing safety surveillance system, the Mini-Sentinel Initiative was launched in 2009, and was eventually expanded to the full Sentinel Initiative in February 2016. The initiative includes electronic data from different healthcare data holders that is automatically collected on an ongoing basis and merged into a common data format, which the FDA can query at any point to quickly and securely monitor drug safety issues. It now includes over 300 million person years of unduplicated data from over 17 different data partnerships. The Mini-Sentinel Initiative (pilot project) identified a 1.5 (95% CI, 0.2–3.2) increased risk of intussusception per 100,000 infants administered the rotavirus vaccination ^15^, highlighting the power of sharing data to identify adverse events that occur at very low rates.

## Lack of data sharing has consequences

There is a considerable lack of data sharing in observational research. This was shown in a recent review of 237 observational studies published in the BMJ from 1 ^st^ January 2015 to 31 ^st^ August 2017. This work found that 63% of studies reviewed during this period did not share the raw data upon which the analyses were conducted ^16^. There are also important examples of large medical record data databases that do not readily share data with external researchers. These include data from the Spanish regional general practitioner database Base De Datos Para La Investigacion Farmacoepedemiologica en Atencion Primaria ( BIFAP) and the Secure Anonymised Information Linkage ( SAIL) databank, which includes primary and secondary care data from the Welsh population.

Lack of data sharing is potentially not without consequence. For example, the Infections in Oxfordshire Research Database (IORD) showed that any apparent aforementioned UK ‘weekend effect’ on mortality arises from patient-level differences at admission ^3, 17, 18^ Data from IORD has been available for many years and if this was openly accessible for researchers to analyse the negative impacts of policy changes could perhaps have been prevented. Rosiglitazone, a glitazone used in the treatment of type II diabetes, was initially approved by the European Medicines Agency in 1999, despite there being limited evidence to support during the approval process. In July 2010, the FDA convened an expert panel to discuss removing rosiglitazone from the market because of arising evidence that rosiglitazone was associated with increasing risk of myocardial infarction. The original evidence was based on the RECORD trial, which was an unblinded, open label trial ^19^. It then took two additional clinical trials and over 10 years of post-marketing surveillance to detect an increased risk in myocardial infarction ^20^. Had a real-world PASS been carried out immediately after market approval utilising a number of different data sources along the lines of the aforementioned Sentinel initiative, unwarranted clinical outcomes could have been robustly detected much earlier.

## Barriers to data sharing; combating privacy concerns

A fundamental barrier associated with the sharing of RWD are the concerns over patient confidentiality. This was illustrated when the UK governmental Care.data programme, which aimed to create a national combined primary and secondary care database, was halted over general public concerns for patient privacy ( https://blogs.biomedcentral.com/on-medicine/2016/08/19/need-care-closure-care-data/). Stricter regulatory frameworks introduced as part of the General Data Protection Regulation ( GDPR) within the EU aim to tackle many privacy issues involved in the storage, processing and management of data in a digital era. New regulations will not only tighten organisational laws around the handling of data, but will also work to increase the rights of individuals, giving them more control over their personal information including the right to transparency, access and erasure. Importantly, despite privacy concerns, studies demonstrate that patients are generally willing to share their healthcare data in the context of contributing to public health as long as the potential benefits are appropriately communicated ^20, 21^

## Conclusion

Even though there are significant potential benefits that improved access to RWD could provide patients, the progress to increase data sharing is slow. There are policy and privacy questions that need to be tackled ^22^, but opening up and sharing healthcare data offers remarkable potential for improvements in care for individuals as well as potential for more effective use of limited healthcare resources. In some instances, for example with the National Quality Registries in Sweden, this potential is already being realised. It is hoped that sharing of RWD becomes standard practice, especially in those data-sets that have a high likelihood of influencing public health and clinical practice.

## Data availability

No data are associated with this article.
