# Supplementary material for: Time to really share real-world data?
Source: F1000Res. 2018 Jul 11;7:1054. [Version 1] doi: 10.12688/f1000research.15517.1 (PMC6192440; doi:10.12688/f1000research.15517.1)
Supplement: Peer review history

# Peer review: Referee response for version 1

**DOI:** 10.5256/f1000research.16919.r38761

**Published:** 2018-10-15

**Authors:** Laurie A. Tomlinson

**Affiliations:** 1 Department of Non‐Communicable Disease Epidemiology, London School of Hygiene & Tropical Medicine, London, UK

Should real-world data be more freely available? Of course it should, it is impossible to disagree. But, "I think you'll find it's a bit more complicated than that".

 Use of observational data for drug safety (among other things) is crucially important. However, many things are critical to make this happen. High quality research training with access to excellent statisticians. A research system that doesn't depend on impact factor and no benefit for getting an 'interesting' result. Quality peer-review. Lots of funding to do important work of this kind. An educated audience capable of critical analysis of complex study designs. Brexit not gutting the MHRA. Close links with the EMA. Affordable access for academics to access the data. And yes open data would help but who has the time to rerun the analyses of other groups who have proven themselves trustworthy when there are probably more important things to do? There are also risks. Anti-vaxxers on a mission to identify spurious drug-effects. And vitally loss of public trust. Yes there are surveys showing public support for access to their data but we have already made a mess of this with care.data and can we risk that again? Especially when pharma or profit-driven companies are promoting it? In a world ever more driven by conspiracy theories and paranoia this seems a big step at the moment.

 Yes real-world data should be more open. But at the moment it is only one of many important battles to fight.

I have read this submission. I believe that I have an appropriate level of expertise to confirm that it is of an acceptable scientific standard.

# Peer review: Referee response for version 1

**DOI:** 10.5256/f1000research.16919.r36415

**Published:** 2018-07-26

**Authors:** David Madigan

**Affiliations:** 1 Department of Statistics, Columbia University, New York City, NY, USA

This is in interesting short opinion piece about RWD. I worry that it exhibits the kind of unbridled enthusiasm that I fear ultimately undermines efforts to appropriately incorporate RWD into healthcare decision making. There are real limitations to RWD studies and the authors should acknowledge these. See, for example, many of Ioannidis’s papers or Madigan, D., et al. (2013) ^1^.

 The CPRD is not a particularly good example since the data are available to researchers under severe restrictions, e.g., “A dataset may only be used for the study for which it has been approved.”

 As I understand it, Sentinel queries require multi-week/month turnaround times.

 The notion that an observational study could have uncovered the rosiglitazone problem early is speculative.

 The section on privacy concerns pays lip service to some really complex issues. These data are de-identified in superficial ways and identification of specific individuals is entirely possible. The implications for health insurance, employment, etc. are highly complex.

 The paper should discuss common data models. Shared data with idiosyncratic data models are of somewhat limited value. In a similar vein, the article should discuss OHDSI.

I have read this submission. I believe that I have an appropriate level of expertise to confirm that it is of an acceptable scientific standard, however I have significant reservations, as outlined above.

## References

[1] MadiganDRyanPBSchuemieMStangPEOverhageJMHartzemaAGSuchardMADuMouchelWBerlinJA: Evaluating the impact of database heterogeneity on observational study results. *Am J Epidemiol*.2013;178(4) : 10.1093/aje/kwt010 645-51 10.1093/aje/kwt010 23648805PMC3736754

# Peer review: Referee response for version 1

**DOI:** 10.5256/f1000research.16919.r35957

**Published:** 2018-07-18

**Authors:** Sajan Khosla

**Affiliations:** 1 AstraZeneca Academy House, Cambridge, UK

I would suggest adding commentary on the reproducibility of RWD studies by better sharing of code and data. Those practitioners of RWD are all aware of the intricate nuances that lead to variable definitions and sharing of these may enable better reproducibility. Perhaps the better use of technology to better develop transparency and reproducibility would be beneficial in these areas, leading the clinical trial setting ^1^.

 The importance of RWD is a combination of the potential regulatory use within the context of the 21st Century Cures act, being able to reinforce clinical decision making with information at the patient bedside and to also support policy making decisions. Having been part of the team on the weekend mortality paper, this analysis was less influential but more supportive to larger initiatives at hand. I would also support that with the outputs from international disease registries in oncology which highlight how poor/late presentation of cancer within the UK system leads to the significant gap in cancer outcomes and led to better public health initiatives around recognising symptoms of cancer ^2^.

I have read this submission. I believe that I have an appropriate level of expertise to confirm that it is of an acceptable scientific standard.

## References

[1] HripcsakGDukeJDShahNHReichCGHuserVSchuemieMJSuchardMAParkRWWongICRijnbeekPRvan der LeiJPrattNNorénGNLiYCStangPEMadiganDRyanPB: Observational Health Data Sciences and Informatics (OHDSI): Opportunities for Observational Researchers. *Stud Health Technol Inform*.2015;216:574-8 26262116PMC4815923

[2] RachetBMaringeCNurUQuaresmaMShahAWoodsLMEllisLWaltersSFormanDStewardJColemanMP: Population-based cancer survival trends in England and Wales up to 2007: an assessment of the NHS cancer plan for England. *Lancet Oncol*.2009;10(4) : 10.1016/S1470-2045(09)70028-2 351-69 10.1016/S1470-2045(09)70028-2 19303813
